# Ultrasonic Effect on the Growth of Crystals from Aqueous Electrolyte Solutions on Polymer Substrates: The Role of Isotopic Composition of Liquid

**DOI:** 10.3390/polym16243580

**Published:** 2024-12-21

**Authors:** Nikolai F. Bunkin, Polina N. Bolotskova, Sergey V. Gudkov, Valery V. Voronov, Vladimir I. Pustovoy, Valery N. Sorokovikov, Oleg T. Kamenev, Yulia V. Novakovskaya

**Affiliations:** 1 Department of Fundamental Sciences, Bauman Moscow State Technical University, 2nd Bauman Str. 5, Moscow 105005, Russia; bolotskova@inbox.ru; 2 Prokhorov General Physics Institute of the Russian Academy of Sciences, Vavilov Str. 38, Moscow 119991, Russia; s_makariy@rambler.ru (S.V.G.); voronov@lst.gpi.ru (V.V.V.); pustovoy@nsc.gpi.ru (V.I.P.); sorokovikov@mail.ru (V.N.S.); 3 Institute of Automation and Control Processes, Far Eastern Branch of the Russian Academy of Sciences, Radio St., 5, Vladivostok 690041, Russia; okamenev@mail.ru; 4 Chemistry Department, Lomonosov Moscow State University, Leninskie Gory, 1/3, Moscow 119991, Russia; jvnovakovskaya@gmail.com

**Keywords:** deionized water, crystallization, aqueous electrolyte solution, polymer membrane, ultrasonic beam, X-ray diffractometry

## Abstract

The peculiarities of the crystal formation from supersaturated aqueous solutions of CuSO_4_ on polymer substrates were studied using X-ray diffractometry. During the crystal formation, the test solutions were irradiated with one or two counter-propagating ultrasonic beams. Test solutions were prepared using natural deionized water with a deuterium content of 157 ± 1 ppm. The other liquid used was deuterium-depleted water with a deuterium content of 3 ppm. It was shown that irradiation with one/two ultrasonic beams resulted in drastic changes in the structure of the crystal deposit formed on the polymer substrate in the case when natural deionized water was chosen for preparing the supersaturated solution of CuSO_4_.

## 1. Introduction

Controlling the crystallization processes of organic and inorganic compounds is a topical scientific problem. The phenomenon of nucleation and growth of crystals has been known for a long time, but has not been fully studied, as noted in recent studies [1,2,3,4]. Crystals with various physical properties are usually obtained by deposition on specially prepared substrates [5,6]. In a recent review [7], the features of crystallization from supersaturated electrolyte solutions on polymer substrates are described. Our study is also devoted to the peculiarities of crystal formation from supersaturated solutions on polymer substrates; a key difference from the works overviewed in [7] is that, in our experiments, the interface between the aqueous solution and the polymer substrate was tangentially irradiated with one ultrasonic beam or two counter-propagating beams. We studied the polymer membrane Nafion™ with the following chemical formula [8]:



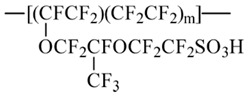

(1)


Nafion N117 specimens, with a fluorocarbon chain parameter m = 6.5, had a thickness of 180 μm. It can be seen from Formula (1) that the Nafion skeleton consists of perfluorinated hydrocarbon chains (C_2_F_4_)_m_ (Teflon) with side perfluorinated ether branches terminated with sulfonic groups SO_3_H. The Teflon matrix exhibited substantial hydrophobic properties, whereas SO_3_H groups were hydrophilic. Because the polymer structure includes a hydrophobic skeleton, hydrophilic terminal SO_3_H groups, and weakly hydrophilic polyether fragments between them, individual segments of the polymer matrix interact with water and aqueous solutions in different ways. This is why, when Nafion swells in water, micelle formation is observed; see Ref. [8] for more detail. In the characteristics of polymer that are revealed upon soaking, a significant role should be played by the dissociation of SO_3_H groups at the water boundary, which is accompanied by the formation of hydronium ions, as follows:

R—SO_3_H + H_2_O ⇔ R—SO_3_^−^ + H_3_O^+^ (2)

As is known (see [8]), during the swelling of Nafion, channels with a diameter of 2–3 nm are formed inside the polymer. The negatively charged inner areas of the channels (in accordance with Reaction (2)) provide conditions for cationic transport through the polymer [9]. The sizes of these channels allow H^+^ and OH^−^ ions to be spatially separated on opposite sides of the membrane; this effect is used in low-temperature hydrogen fuel cells [10,11]. The ion-exchange properties and morphology of Nafion expand the area of its application. Indeed, Nafion membrane can be used as a matrix for creating composite structures by introducing various chemicals into its volume, for example, methylene blue cationic dye [12,13], as well as colloidal nanoparticles [14]. In addition, our recent works describe experiments where Nafion substrates played the role of a template in the deposition of crystals from supersaturated solutions of various electrolytes [15,16].

Photoluminescence spectroscopy techniques are actively used to study the physical processes on the surface of Nafion (see Refs. [17,18]). In particular, it was found that the state of the polymer-water interface is controlled by deuterium content. It was found that when the polymer swells in water, the reorganization of the subsurface polymer fibers is probably accompanied by different extents of effective unfolding of their side chains into the water phase, depending on the content of deuterium in it. In experiments on photoluminescence spectroscopy [17,18], it was shown that the effect of unfolding is most noticeable when the polymer is soaked in water with the content of deuterium in the range of 10^2^–10^3^ ppm (obviously, the deuterium content in natural water, which is equal to 157 ± 1 ppm, falls in this range) and is not manifested when the polymer swells in deuterium-depleted water (DDW; the content of deuterium is ≤3 ppm).

According to the data of atomic force microscopy and low-angle X-ray scattering in the geometry of grazing incidence [19], the subsurface micelles of Nafion polymer fibers, being in contact with natural water, are predominantly oriented normally to the Nafion-water interface, and the mean distance between fibers of these micelles can be of an order of nanometers. According to theoretical estimates given in our recent works [15,16], the distance between the neighboring side chains terminated with sulfonate groups can be as small as 13–15 Å when all the chains are unfolded. The distances are comparable to (or slightly exceed) the characteristic size of unit cells of many crystal lattices.

The X-ray diffractometry used in [15,16] enables the study of the specific features of crystal deposition from supersaturated aqueous electrolytic solutions on a Nafion polymer substrate. These solutions were prepared using natural water (NW and NW-solutions) and using deuterium-depleted water (DDW and DDW-solutions, respectively). Partial unfolding of the polymer chains was found to affect crystal formation when the Nafion membrane was used as a substrate. This effect is manifested in different ways and depends on the crystal lattice and the deuterium content of the water, on the basis of which the electrolyte solution was prepared. If we deal with NW-solution (where the effect of polymer unfolding occurs), then the polymer chains on the Nafion surface affect the crystallization of various substances in different ways. Particularly, in the case of the cubic lattice of KCl and the monoclinic one of sodium acetate trihydrate NaCH_3_COO × 3H_2_O, no difference was found in the diffraction patterns of crystals deposited on polymer substrates from supersaturated NW-solutions and DDW-solutions. In the case of copper sulfate, whose crystals can be either pentahydrates CuSO_4_ × 5H_2_O (triclinic syngony) or trihydrates CuSO_4_ × 3H_2_O (monoclinic syngony), CuSO_4_ trihydrate is deposited on the polymer substrate from a supersaturated NW-solution of CuSO_4_. At the same time, a triclinic syngony of the CuSO_4_ × 5H_2_O pentahydrate deposit was always identified in the case where the supersaturated solutions were prepared using DDW.

As shown in [15], unfolded polymer side chains can affect the syngony of CuSO_4_ crystals because of the following peculiarities: in the case of the triclinic syngony of CuSO_4_ × 5H_2_O pentahydrate, the angles between the translation vectors of the unit crystal cell are α = 77.33°, β = 82.27°, and γ = 72.57°, indicating that there is no orthogonality between the lattice vectors (the unit crystal cell is a skewed parallelepiped), whereas for the monoclinic syngony of CuSO_4_ × 3H_2_O trihydrate, the angles are α = 90°, β = 97.1°, and γ = 90°, i.e., the unit crystal cell is a rectangular parallelepiped (crystallographic data are taken from [20]). In addition, the distance between the terminal sulfonic groups of the polymer chains, when the latter are completely unfolded normally to the surface of the membrane, is a multiple of one of the edges of the crystal lattice cell. Thus, the polymer fibers are complementary to the geometry of the cell and can assist in the formation of CuSO_4_ trihydrate on the polymer substrate.

Here, we continue the investigations of the crystal growth from supersaturated aqueous solutions of CuSO_4_ on a polymer substrate. In these experiments, an aqueous solution of CuSO_4_ was irradiated with one or two counter-propagating ultrasonic beams during the formation of a deposit on a polymer substrate. As was shown in our recent work [21], the state of unfolded polymer chains can be affected by ultrasonic beams directed tangentially to the Nafion-liquid interface across the unfolded polymer fibers. The mechanism of ultrasonic effect on unfolded chains is due to the absorption of an ultrasonic beam close to the polymer membrane in the near-surface area of the solvent. The absorption of ultrasound leads to the generation of acoustic flows along the direction of the original ultrasonic beam propagation. These flows efficiently shift the ends of the polymer chains so that they are no longer directed normally to the mean polymer surface; see [21]. Therefore, it can be expected that ultrasonic irradiation can be used to control the local microstructure of crystals deposited on a polymer substrate. We wanted to check whether fibers unwound from the surface of a polymer substrate and oriented perpendicular to the substrate can affect the syngony of the crystalline deposit on the polymer substrate and whether this syngony can be controlled using one or two counter-propagating ultrasonic waves. It is clear that CuSO_4_ is most suitable for this purpose, since the elementary cell of this crystal is a rectangular parallelepiped in the case of CuSO_4_ trihydrate and a skewed parallelepiped in the case of CuSO_4_ pentahydrate.

The goal of this work was to study the specific features of the growth of CuSO_4_ crystals from supersaturated aqueous solutions with different isotopic compositions on polymer substrates under the effect of concurrent ultrasound irradiation; the corresponding analysis is based on X-ray diffraction experiments. The novelty of our research lies precisely in the study of the possibility of controlling the syngony of a crystalline deposit on a polymer substrate using ultrasonic wave irradiation.

## 2. Materials and Methods

In our experiments, a 180 μm thick Nafion N117 membrane (Sigma Aldrich, St. Louis, MO, USA) was used. The Nafion plates were soaked in the test liquids. One of the test liquids was deionized natural water (deuterium content is equal to 157 ± 1 ppm) with a resistivity of 18 MΩ cm at 25 °C. Samples of this water were obtained with a Milli-Q apparatus (Merck KGaA, Darmstadt, Germany). The other test liquid was deuterium-depleted water (DDW) with a deuterium content of ≤3 ppm, and D_2_O (99.9 atom % D), purchased from Sigma Aldrich, St. Louis, MO, USA.

The protocol for preparing liquid samples was as follows: 22.3 g of anhydrous crystalline CuSO_4_ (Sigma Aldrich, St. Louis, MO, USA; chemical purity meets analytical specification of Ph. Eur., BP, USP, 99–100.5%, based on anhydrous substance) was dissolved in 100 mL of NW or DDW; this concentration corresponds to a saturated solution at *T* = 25 °C. The resulting liquid samples were poured into open (not isolated from the atmosphere) optical glass cuvettes measuring 24 × 40 × 50 mm^3^. The growth of CuSO_4_ crystals occurred at a constant temperature, which was ensured by placing the cuvette in a closed foam box.

The crystals were deposited on a Nafion polymer substrate, which was placed on the bottom of the cuvette. In a series of experiments, the cuvette with the solution was irradiated with ultrasonic beams using one or two piezoceramic transducers attached outside the cuvette near the bottom along its longer face (50 mm); when using two piezoelectric elements, the ultrasonic beams were directed toward each other. Ultrasonic irradiation was generated by round piezoelectric transducers (∅ = 8 mm). The piezoelectric transducers were made of piezoceramics Pb(Zr*_x_*Ti_1-*x*_)O_3_; here *x* means the mole fraction, *x* ≈ 0.48. The thickness of the piezoelectric transducer (piezoelectric element) is *L*_0_ = 350 μm. Assuming that the speed of longitudinal sound in the ceramics used is equal to *C_sound_* = 4.32 × 10^3^ m/s (see monograph [22]), and taking into account *L*_0_ = λ/2, where λ is the wavelength of longitudinal sound, we obtain that the frequency of sound waves ν = 6.1 MHz.

**Figure 1:**
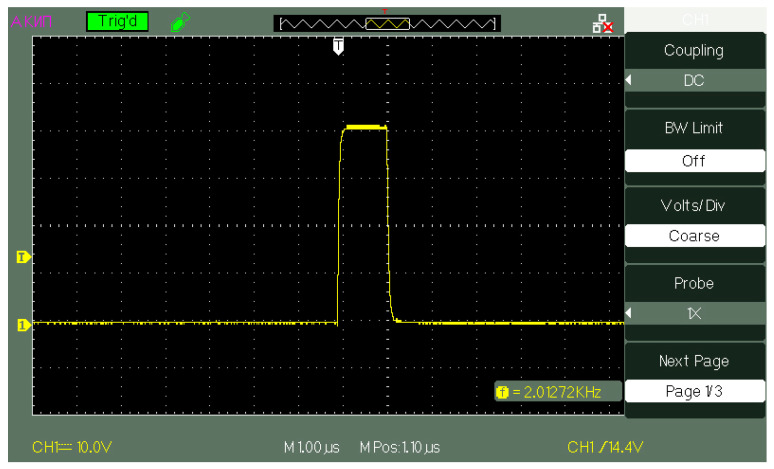
Oscillogram of electrical pulses supplied to a piezoelectric transducer.

A G5-63 pulse generator (Priborelectro, Moscow, Russia) was used to produce ultrasonic waves. The ultrasonic signals from the piezoelectric transducers were monitored using an AKIP 4115/3A digital oscilloscope (Novapribor, Moscow, Russia). Pulse voltage with variable amplitudes and a duration of 1 μs was supplied to the transducers. Figure 1 shows an oscillogram of a pulse, having a duration of 1 μs and an amplitude of *U*_0_ = 40 V. The pulse repetition frequency was fixed and amounted to 2 kHz.

X-ray patterns of the crystals were obtained with a Bruker D8 Discover A25 DaVinci (Billerica, MA, USA) Design diffractometer using the Debye–Scherrer method. The radiation source was a Siemens KFL ceramic X-ray tube, having the focal size 0.4 × 12 mm^2^. Shooting modes were the following: CuKα radiation, Kβ filter, *U* = 40 kV, *I* = 40 mA. We used Bragg-Brentano geometry with Soller collimators of 2.5° and slit width of 0.638 mm. The LYNXEYE detector was used; the angular interval was 2θ = (10–65)° with a scanning step of 0.01°; the exposure time at each step was 7.5 s. The diffraction patterns were processed by the EVA program, version 2.1. The identification of X-ray reflexes was performed with the use of the PDF-2 database [23].

In order to elucidate the effect of the isotopic composition on the unwinding of polymer fibers, 100 mL solutions of liquid samples with deuterium content of 100 ppm, 500 ppm, 10^3^ ppm, 10^4^ ppm, and 10^5^ ppm were prepared via volumetric mixing of DDW and D_2_O. Then, a Nafion plate with a size of 15 × 20 mm^2^ was immersed in the bottom of the cell of height 60 mm, containing these liquids, DDW and D_2_O. After that, the pH value vs. the deuterium content of the sample for different soaking times of the Nafion plate was measured. Here, we used a Mettler Toledo Seven Excellence pH meter S400-uMix-Kit (Mettler-Toledo Philippines Inc., Muntinlupa, 1780, Philippines); the pH meter sensor was immersed in the bottom of the cell.

To interpret the experimental results, we performed non-empirical quantum-chemical calculations and simulations. To describe a segment of the main chain of the Nafion polymer with two side chains terminated each with a sulfonic group, the following model structure was constructed:

HSO_3_CF_2_CF_2_OCF(CF_3_)CF_2_O—CF(CF_3_)(CF_2_)_14_CF(CF_3_)—OCF_2_CF(CF_3_)OCF_2_CF_2_SO_3_H

Locally stable structures of this fragment, which differ in the configuration and mutual arrangement of the side chains, were found in the gas phase and in the presence of 20–35 water molecules. The calculations were performed in the density functional approximation with the use of the B3LYP hybrid exchange-correlation functional [24,25,26,27] (which correctly describes both the majority of functionalized organic molecules and systems stabilized by hydrogen bonds) and the extended double-zeta basis set of 6–31G(d,p) quality [28,29,30]. To take into account the dispersion interactions, which are important in the case of perfluorinated domains of the structures under study, the Grimme D3 correction [31] with the Becke–Johnson modification [32] was used. This approach correctly reproduces the electron density distribution of both substituted hydrocarbons and water clusters owing to the balanced description of the effects of hydrogen bonds together with electrostatic and dispersion forces. The calculations were carried out with the use of Firefly software package, version 8.2.0. [33], partly based on the GAMESS US code [34]. The Chemcraft program, version 1.8 build 688 [35] was used to visualize the cluster structures.

## 3. Experimental Results

**Figure 2:**
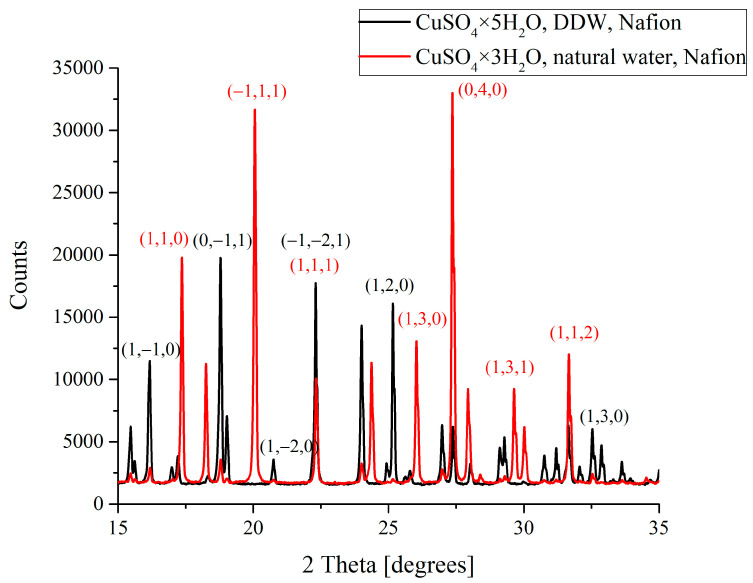
Diffraction patterns of CuSO_4_ crystals deposited on a polymer substrate from DDW-solution (CuSO_4_ × 5H_2_O, black curve) and NW-solution (CuSO_4_ × 3H_2_O, red curve) in the absence of ultrasonic treatment. A set of crystallographic indices (h,k,l) for some of the highest peaks is given; the indices highlighted in red refer to the trihydrate, whereas the indices highlighted in black refer to the pentahydrate.

Figure 2 exhibits the X-ray diffraction patterns of CuSO_4_ crystals deposited on a Nafion substrate from NW-solutions and DDW-solutions in the absence of ultrasonic treatment. As follows from Figure 2 (black curve), if a supersaturated CuSO_4_ solution was prepared using DDW, copper sulfate pentahydrate CuSO_4_ × 5H_2_O with a triclinic syngony is deposited on the polymer substrate. At the same time, if we deal with supersaturated NW-solution, copper sulfate trihydrate (CuSO_4_ × 3H_2_O, monoclinic syngony) grows on the polymer substrate. It is worth noting that, the other conditions being the same, the triclinic syngony is energetically more favorable. As shown in [15,16], the deposition of crystals with monoclinic syngony on the polymer substrate is due to the spatial complementarity of one of the crystallographic directions of copper sulfate trihydrate and the arrangement of sulfonic groups at the ends of unfolded Nafion side chains. In this figure, a set of crystallographic indices (h,k,l) for some of the highest peaks is given; the indices highlighted in red refer to the trihydrate, and the indices highlighted in black are related to the pentahydrate; these data were taken from [23].

**Figure 3:**
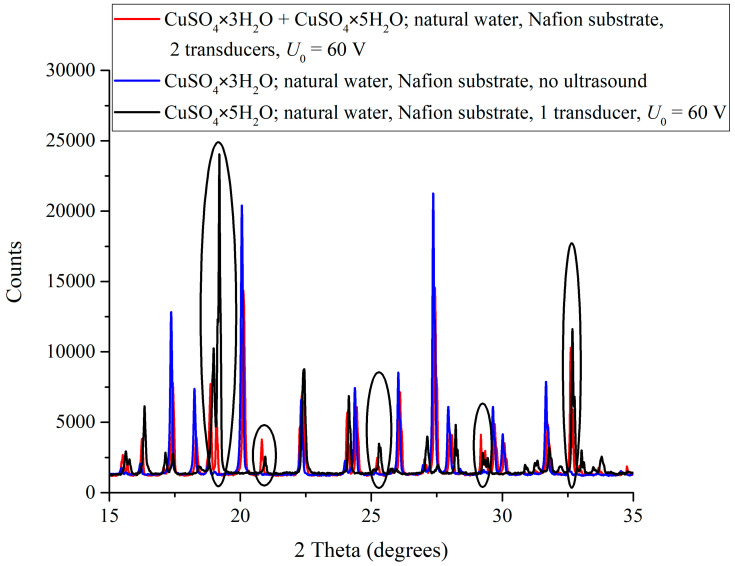
Diffraction patterns of CuSO_4_ crystal hydrates grown on a polymer substrate from NW- solution under irradiation with a single ultrasonic beam at a pulse voltage on the piezoelectric element of *U*_0_ = 60 V (black curve), under irradiation with two counter-propagating ultrasonic beams at a pulse voltage on the piezoelectric elements of *U*_0_ = 60 V (red curve), and in the absence of irradiation (blue curve). Black ovals mark reflexes related to the structure of CuSO_4_ × 5H_2_O pentahydrate included in the trihydrate matrix as a result of ultrasonic treatment.

Figure 3 shows the diffraction patterns of CuSO_4_ crystals formed on a polymer substrate from a supersaturated NW-solution under the ultrasonic irradiation of the “polymer membrane—liquid” interface with the use of a single source at a pulsed voltage on the piezoelectric element of *U*_0_ = 60 V (black curve), as well as upon irradiation with two counter-propagating ultrasonic beams at a pulsed voltage on the piezoelectric elements of *U*_0_ = 60 V (red curve) and in the absence of ultrasonic treatment (blue curve). In the absence of irradiation, CuSO_4_ × 3H_2_O trihydrate is deposited on the polymer substrate, whereas upon irradiation with a single ultrasonic beam, CuSO_4_ × 5H_2_O pentahydrate is formed on the polymer substrate; upon irradiation with two counter-propagating ultrasonic beams, a mixture of tri- and pentahydrate is deposited on the substrate (CuSO_4_ × 3H_2_O+ CuSO_4_ × 5H_2_O, red curve). In the figure, black ovals mark the spectral reflexes related to CuSO_4_ × 5H_2_O pentahydrate grown as inclusions in the trihydrate crystal matrix as a result of ultrasonic treatment.

**Figure 4:**
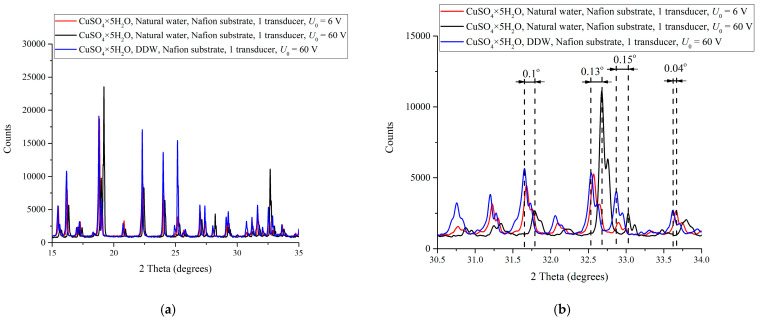
X-ray diffraction patterns of CuSO_4_ crystal hydrates deposited on the Nafion substrate from NW-solution under irradiation with a single ultrasonic beam at a pulsed voltage on the piezoelectric element of *U*_0_ = 60 V (black curve) and *U*_0_ = 6 V (red curve), as well as deposited on the Nafion substrate from the DDW-solution at a pulsed voltage of *U*_0_ = 60 V (blue curve). Panel (**a**) shows the reflexes in a range of 15–35°. Panel (**b**) shows the reflexes in a range of 30.5–34°.

Figure 4a,b show the diffraction patterns of CuSO_4_ crystal hydrates deposited on Nafion substrate from NW-solution under irradiation of the “polymer membrane—liquid” interface with a single ultrasonic beam at a pulsed voltage on the piezoelectric element of *U*_0_ = 60 V (black curve) and under irradiation with a single ultrasonic beam at *U*_0_ = 6 V (red curve), as well as CuSO_4_ crystal hydrates deposited on Nafion substrate from the DDW-solution under irradiation with an ultrasonic beam at *U*_0_ = 60 V (blue curve). Note that when crystal was deposited from a DDW-solution, the “polymer membrane—liquid interface” was also irradiated with one ultrasonic beam at *U*_0_ = 6 V, but the diffraction pattern obtained in this case does not differ from the pattern at *U*_0_ = 60 V. 

As follows from Figure 4, in all cases, CuSO_4_ × 5H_2_O pentahydrate is formed. In this case, panel (a) shows the general diffraction pattern in a range of angles of 15–35°, and panel (b) shows a magnified pattern in a range of 30.5–34°. It is evident that the reflexes of the deposit formed on the polymer substrate from NW-solution are shifted in the case of ultrasonic irradiation with one beam by the value Δθ to the larger angles compared to those of the deposit obtained from the DDW-solution. As follows from the graphs of panel (b), with an increase in the pulse voltage, this shift increases: at *U*_0_ = 6 V, the value Δθ≈ 0.04°, whereas at *U*_0_ = 60 V, Δθ≈ 0.1°–0.15°. Recall that in the absence of ultrasonic irradiation (pulse voltage *U*_0_ = 0), the trihydrate CuSO_4_ × 3H_2_O is deposited on the polymer substrate, i.e., in this case we deal with a crystal of another syngony; see the red pattern in Figure 2.

**Figure 5:**
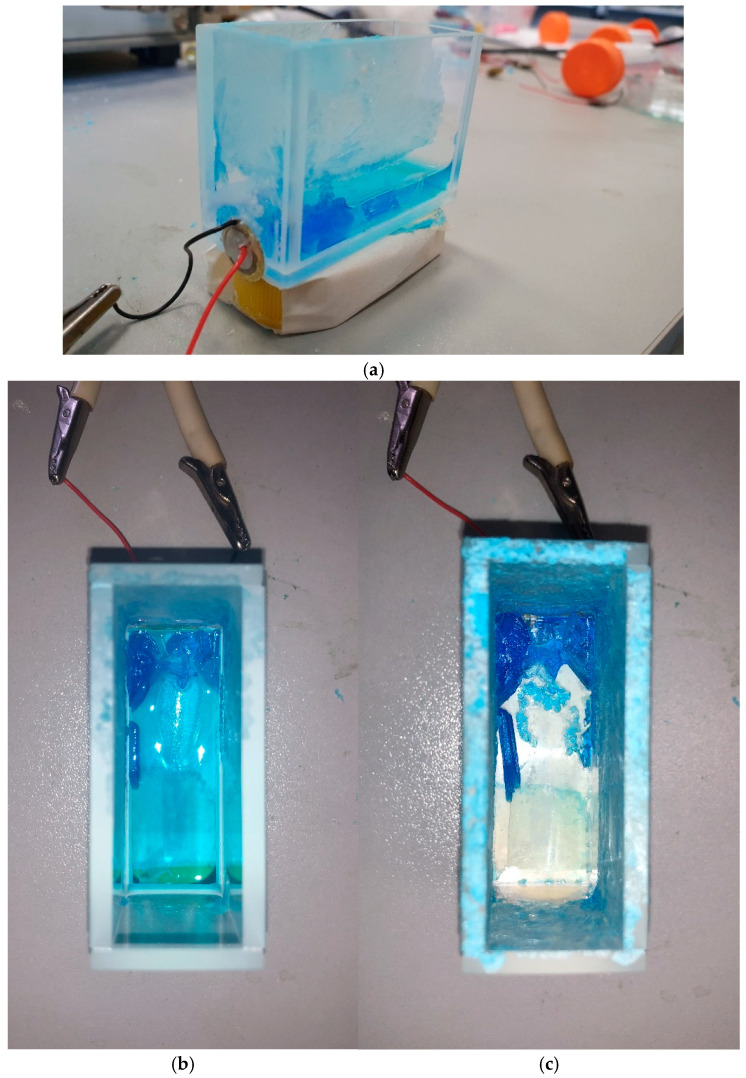
CuSO_4_ crystal hydrate formed on the Nafion substrate from NW-solution under the irradiation with one ultrasonic beam at a pulse voltage of *U*_0_ = 6 V. Panels (**a**,**b**): the formation of the deposit continues, since the solution has not completely evaporated. Panel (**c**): the end of the formation of the deposit; water is absent in the cuvette. It is seen that the deposit is formed predominantly near the piezoelectric transducer.

Figure 5a–c show photographs of CuSO_4_ crystals formed on the Nafion substrate from NW-solution under ultrasonic treatment with the use of one piezoelectric element (pulse voltage on the piezoelectric element *U*_0_ = 6 V). Panels (a) and (b) illustrate the growth of crystals at the stages of incomplete evaporation of water (the crystals are immersed in the solution), and panel (c) corresponds to the end of the formation of the crystal deposit. It is evident that the crystal deposit is formed predominantly in the region near the piezoelectric transducer.

**Figure 6:**
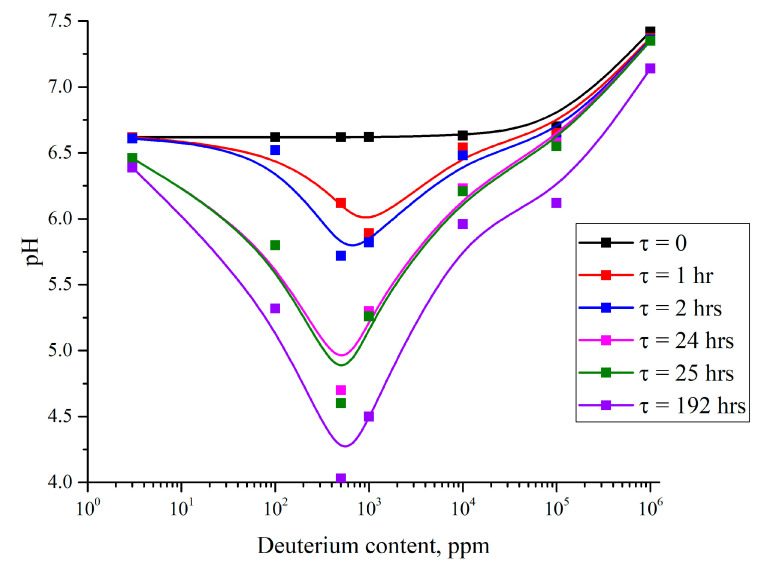
The dependences of pH vs. deuterium content of water; the parameter of these dependences is the soaking time τ. Explanations are in the text.

Figure 6 shows the results of pH measurements depending on the deuterium content in DDW (3 ppm) and D_2_O (10^6^ ppm) samples, as well as in their bulk mixtures with deuterium content of 100 ppm, 500 ppm, 10^3^ ppm, 10^4^ ppm, and 10^5^ ppm. pH measurements were made in liquid samples poured into a cell of 100 mL with height of 60 mm. Nafion plate with an area of 15 × 20 mm^2^ was immersed in the bottom of the cell with liquid. pH measurements were carried out depending on the soaking time τ of the Nafion plate in the test liquid. The time τ = 0 is related to the measurement performed before immersion of the Nafion plate in the cell. Other measurements were carried out for τ = 1 h, 2 h, 24 h, 25 h, and 192 h. As follows from the obtained graphs, the pH value decreases as Nafion is soaked, which is basically explained by formula (2); the liquid becomes more acidic upon swelling. However, the pH value depends non-monotonically on the deuterium content: the strongest changes occur in the range of 100–10^4^ ppm (recall that the deuterium content in natural water is 157 ppm). The obtained curves correlate very well with the dependence on the size of the liquid region occupied by unwound polymer fibers vs. the deuterium content in this liquid; see [17,18]. It follows from the graphs of Figure 6 that the effect of the isotopic composition of liquid on the unwinding of polymer fibers toward the liquid bulk is most likely of a chemical nature. The next section presents a model that qualitatively describes the effect of isotopic composition on the structure of a crystalline deposit on a polymer substrate, taking into account the unwinding of polymer fibers.

## 4. Discussion

Deposition of copper sulfate on the Nafion substrate under treatment with one or two counter-propagating ultrasonic beams shows that ultrasound can be used to control the crystallization process. As already noted, copper sulfate can crystallize in a trihydrate with a monoclinic lattice and a pentahydrate with a triclinic lattice. In the case of trihydrate, the first shell of any copper atom involves three water molecules and three sulfate groups. The distances between the copper and oxygen atoms are not equal and range from 1.96 to 1.98 Å for oxygen atoms of H_2_O molecules and from 1.96 to 2.45 Å for the atoms of the sulfate group. If we conditionally choose the SO_4_–Cu–SO_4_ sequence as the local basic chain (with Cu–O distances of about 2.4 Å), then in the plane, normal to this sequence, three oxygen atoms of three water molecules and one oxygen atom of a copper sulfate form a distorted square with Cu–O distances of 1.96–1.98 Å. Thus, the sulfate groups, located in the first shell of the copper ion, are not completely equivalent. In the case of pentahydrate, the first shell of the copper atom involves four molecules of water and two SO_4_ groups. If we take into account only the positions of the oxygen atoms of water molecules (neglecting the orientation of these molecules), we can claim that the sulfate anions are predominantly located along the local C_4_ symmetry axis. We can assume that the local O-symmetry of the close neighborhood of the copper atom corresponds to an elongated octahedron. Indeed, two Cu…O distances to the sulfate anions are the same and equal to 2.41 Å, whereas the Cu…O distances between the oxygen atoms of the water molecules in opposite pairs are equal with a small difference between these pairs, amounting to 1.97–1.94 Å. Very high local symmetry of the pentahydrate imposes more rigorous restrictions on the structure of the template formed that would rather promote the formation of this crystal hydrate. At the same time, the trihydrate structure of low symmetry, which is characterized by strong variations in the orientation of the SO_4_ sulfate groups with respect to the copper ion, allows this ion to fit more easily into the local domain restricted by the external template, providing that the nature of the boundaries is complementary to the deposit constituents. Particularly, the distance between these boundaries (terminated with sulfonic groups) should correspond to (to be multiples of) one of the basic distances between the sulfate groups SO_4_ in the copper hydrate. As was found as a result of quantum chemical simulations of the model structures [15], the distance between uniformly unfolded Nafion side chains at their mean normal orientation with respect to the perfluorinated Teflon framework can be 11–13 Å, which correlates well with the distances in the CuSO_4_ trihydrate.

**Figure 7:**
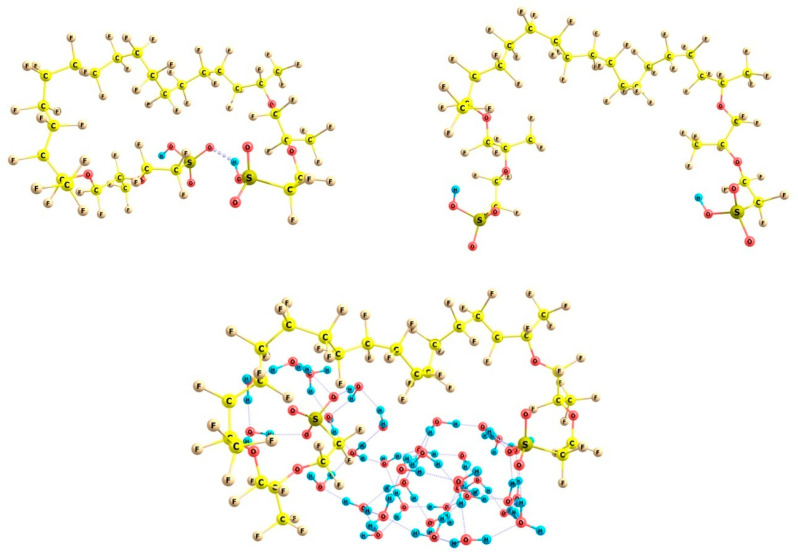
Locally stable configurations of a model fragment of the Nafion polymer matrix with folded (**left**), unfolded (**right**), and partially unfolded and hydrated side polymer chains in the presence of 35 water molecules (**bottom**).

It should be noted that in the dry (anhydrous) state of Nafion, the formation of hydrogen bonds between the sulfonic groups of the adjacent side chains is energetically favorable. Figure 6 shows two configurations of a model fragment of the Nafion polymeric matrix, which involve completely folded and substantially unfolded side chains. The configuration, which involves folded chains, is stabilized by a hydrogen bond between the terminal SO_3_H groups and has an energy that is 19 kcal/mol lower than that of the configuration with unfolded chains. But, in the presence of water molecules, the situation drastically changes. As was shown in [15], the formation of even the first hydration shell composed of 16 water molecules around one sulfonic group leads to its dissociation and lowers the energy of the system by 44 kcal/mol compared to the situation where a cluster of water molecules is localized close to a single-chain fragment of Nafion. However, the process of hydration of the side polymer chain, which is spontaneous in the case of a single chain, is characterized by a high energy barrier in the case of the initially folded and bound neighboring chains, since in this case the repulsion between the hydrophobic segments of the polymer and water molecules is dominant. Additionally, there should be forces that govern the unfolding. Only a noticeable perturbation of the polymer chains, which disturb the hydrophobic contacts and initiate the restricted local rotations of the backbone functional groups with respect to each other, can promote the unfolding; the consistent internal rotations and inclusion of water molecules in between the chains can lead to the progressive unfolding, which ends up with the extension of the chains nearly normally to the polymer backbone and their stable separation with water molecules, as illustrated by the bottom structure in Figure 7.

If the polymer chains located on the surface of the Nafion substrate are unfolded toward the liquid bulk (this is possible if the polymer swells in natural water), then, as was shown in our recent work [21], the bulk and shear viscosity of the near-surface layer, with a thickness that exceeds the length of the unfolded chains (this size is about 15 Å), increases significantly due to the structuring of the outer water molecules. If an ultrasonic beam propagates inside such a layer in the direction normal to the unfolded polymer chains, this beam is absorbed, and the wave completely attenuates. In this case, due to the law of momentum conservation, the momentum of the sound wave must be transferred to the particles of the medium, i.e., an acoustic flow arises in the direction of the initial ultrasonic wave; see, for example, [36,37,38,39]. In [21], we investigated the effect of such hydrodynamic flows upon Nafion swelling in experiments with photoluminescence spectroscopy; the modes of excitation of such flows were studied when a polymer membrane was treated with one and two ultrasonic waves. It was shown that in the case of irradiation with one ultrasonic beam, polymer chains, initially oriented normally to the polymer surface, are efficiently deflected by such a flow so that the angle between the chain and the substrate becomes less than 90°. At the same time, when two opposite and identical in intensity acoustic flows are excited, the polymer chains should oscillate around the mean orthogonal orientation with respect to the polymer surface. Such a model enables one to suggest a qualitative explanation of the features of the formation of CuSO_4_ crystal hydrates revealed by the X-ray structure analysis, which were presented in the previous section.

In the absence of ultrasonic treatment (Figure 2), CuSO_4_ × 3H_2_O trihydrate with a monoclinic lattice is formed on the polymer substrate from the supersaturated NW-solution. In this case, the angles between the translation vectors of the elementary cell are α = 90°, β = 97.1°, and γ = 90°, that is, the elementary cell is a rectangular parallelepiped. Such an orientation of the translation vectors is complementary to the polymer chains unfolded normally to the substrate surface. At the same time, in the CuSO_4_ DDW-solution, the unfolding effect is absent, and therefore the polymer substrate cannot play the role of a structuring template, and pentahydrate CuSO_4_ × 5H_2_O with triclinic syngony is formed. Recall that the deposition of pentahydrate is energetically more favorable; see above.

When NW-solution is irradiated with a single ultrasonic beam in the direction normal to the unfolded polymer chains, acoustic flows are generated, which decreases angles between the unfolded chains terminated by sulfonic groups and the mean polymer surface. Moreover, under such conditions, one should expect variations (both spatial and temporal) in the distances between the ends of the polymer chains due to their different phase shifts relative to the maxima of the ultrasonic wave. Taking into account the characteristic times of crystal hydrate formation, such spatial-temporal averaging of positions of the polymer chains should be revealed in the coordinating effect of the polymer substrate.

In the case of one ultrasonic beam, the deposition of CuSO_4_ × 5H_2_O pentahydrate becomes more probable; see Figure 3 and Figure 4a,b. In this case, in the region of the solution near the piezoelectric element, where the effect of ultrasound is most significant due to the destruction of the hydrogen-bond network in the solvate shells of ions, the most favorable conditions for the crystal deposition are formed, which is shown in the photographs Figure 5a–c. It is also noteworthy that, when the specimen is irradiated with one ultrasonic beam, the reflexes in the diffraction patterns of the deposits are shifted toward larger angles compared to the crystal deposit on the polymer substrate obtained from a supersaturated DDW-solution also irradiated with one ultrasonic beam, and the Δθ value of the shift changes with a change in the amplitude of the pulse voltage on the piezoelectric element. Thus, at the pulse voltage on the piezoelectric element *U*_0_ = 6 V, we have Δθ ≈ 0.04°, whereas at *U*_0_ = 60 V, Δθ ≈ 0.1°–0.15° (see Figure 4a,b). Such shifts in combination with small changes in the width and profile of the reflexes as a whole may indicate certain changes in the inter-planar distances and a disturbance of the translation vector in the direction normal to the substrate. Recall that the structure of the crystal deposit obtained from the supersaturated DDW-solution does not depend on the amplitude *U*_0_ of the pulse voltage on the piezoelectric element.

At the same time, when the specimen was irradiated with two counter-propagating ultrasonic beams, the diffraction patterns of the deposits correspond to the CuSO_4_ × 3H_2_O trihydrate with a monoclinic lattice. This was to be expected; in this case, the mean orientation of the unfolded polymer chains normal to the surface of the polymer backbone is preserved; see [21]. As shown in Figure 4, in this case, the diffraction pattern also contains a number of reflexes related to CuSO_4_ × 5H_2_O.

Here, we would like to emphasize that we used very low intensities of the sound wave. The corresponding estimates of the sound energy are given in our previous work [22]. As follows from the estimates obtained in [22], the amplitude of the sound wave is *A* = 6 Å, i.e., for the frequency ν = 6.1 MHz (see the Section 2 of this work), the velocity of particles in such a wave is *v* = 2πν*A =* 2.3 × 10^−2^ m/s. Such ultrasonic waves can hardly destroy the polymer membrane. Indeed, in order for the polymer membrane to be destroyed as a result of such an ultrasonic impact, the velocity of Nafion particles inside the polymer bulk must be comparable to the speed of sound in Nafion (the basic criterion for the occurrence of a shock wave that destroys materials, see, for example, [38]). As shown in [40], the speed of longitudinal sound inside the Nafion membrane depends on the water content in the membrane volume and on the temperature, but on average, this speed is about 1500 m/s, much greater than 2.3 × 10^−2^ m/s; see Figure 3 of the study [40].

Note, finally, that the pulse repetition frequency in our experiments did not change and was equal to 2000 Hz. Since the ultrasonic wave must be absorbed in the layer of polymer fibers unwound in the volume of liquid, it is not very important for us whether the ultrasonic wave will exist in the intervals between ultrasonic pulses. It is important for us, however, that hydrodynamic flows are preserved in the intervals between pulses. It is clear, however, that in the future, we should conduct experiments not only for different voltages applied to the transducers but also for different pulse repetition rates.

Concluding, we want to draw attention to the graph illustrating the dependences of the pH of the water in which the Nafion plate is being soaked and the deuterium content in this water; see Figure 6. The parameter of these dependences is the soaking time τ. It is quite obvious that when Nafion is soaked in water, the liquid becomes acidified, i.e., the pH value drops; see formula (2). Here, we have found that the decrease in pH depends non-monotonically on the deuterium content. This means that isotopic composition can play a certain role not only in the processes of crystal deposition on a polymer substrate but in traditional chemical reactions in general. We can thus assume that the effect of unwinding of polymer fibers is most likely of a chemical nature.

## 5. Conclusions

The main conclusion of our studies is the possibility of controlling the structure of the crystalline deposit by irradiating the “polymer substrate–aqueous solution” interface with one or two ultrasonic waves. The deuterium content in the aqueous media is 3 ppm and 157 ppm, i.e., we are talking about negligible concentrations of deuterium, at which the crystalline deposit has a different syngony. As far as we know, experiments in such a setting were performed for the first time. In our work, it was found that in the case of irradiating the “polymer substrate–NW-solution” interface with one ultrasonic wave, copper sulfate pentahydrate is deposited on the substrate, whereas in the absence of irradiation, copper sulfate trihydrate is deposited. Thus, by using very weak ultrasonic exposure, we can change the syngony of the crystalline deposit. The higher the amplitude of the pulse voltage on the piezoelectric element (i.e., the higher the contribution of ultrasound energy during crystal deposition), the greater the distance Δθ between the X-ray reflexes, i.e., the more the structure of the elementary crystal cell of the pentahydrate is distorted. This paper also presents the results of numerical modeling based on the density functional method, which demonstrate at a semi-quantitative level the possibility of controlling the syngony of the crystal deposited on a polymer substrate using ultrasound waves. To our knowledge, these results were obtained for the first time.

Concluding, a polymer substrate can act as a structuring template. In the case of Nafion, the ability to be such a template is predetermined by the unfolding of the side polymer chains terminated by sulfonic groups. Experiments show that the degree (and overall probability) of unfolding of the polymer chains depends on the isotopic composition of water. The mechanism of the effect of ultrasonic beams is due to the absorption of ultrasound in the near-surface solvent layer that involves unfolded polymer chains, and the acoustic flows are generated normally to the chains. These flows can be used to control the amplitude of variations in the angle of inclination of the unfolded chains relative to the surface of the polymer substrate and the degree of destruction of the hydrogen-bond network in the aqueous solution, which promotes changes in the prevailing syngony of the CuSO_4_ crystal hydrate.

## Data Availability

The original contributions presented in this study are included in the article. Further inquiries can be directed to the corresponding author.
